# Phylogenomic and Structural Analysis of the Monkeypox Virus Shows Evolution towards Increased Stability

**DOI:** 10.3390/v15010127

**Published:** 2022-12-31

**Authors:** Priya Yadav, Yashas Devasurmutt, Utpal Tatu

**Affiliations:** Department of Biochemistry, Indian Institute of Science, Bangalore 560012, India

**Keywords:** monkeypox virus, mutation rate, phylogenomics, high mutation, protein structure

## Abstract

Monkeypox is an infectious zoonotic disease caused by an Orthopoxvirus and results in symptoms similar to smallpox. In a recent outbreak, monkeypox virus (MPXV) cases have been reported globally since May 2022, and the numbers are increasing. Monkeypox was first diagnosed in humans in the Democratic Republic of Congo and has now spread to throughout Europe, the USA, and Africa. In this study, we analyzed the whole genome sequences of MPXV sequences from recent outbreaks in various countries and performed phylogenomic analysis. Our analysis of the available human MPXV strains showed the highest mutations per sample in 2022 with the average number of mutations per sample being the highest in South America and the European continents in 2022. We analyzed specific mutations in 11 Indian MPXV strains occurring in the variable end regions of the MPXV genome, where the mutation number was as high as 10 mutations per gene. Among these, envelope glycoproteins, the B2R protein, the Ankyrin repeat protein, DNA polymerase, and the INF alpha receptor-like secreted glycoprotein were seen to have a relatively high number of mutations. We discussed the stabilizing effects of the mutations in some of the highly mutating proteins. Our results showed that the proteins involved in binding to the host receptors were mutating at a faster rate, which empowered the virus for active selection towards increased disease transmissibility and severity.

## 1. Introduction

The name monkeypox originated after the first identification of this virus in monkeys in 1958 in a Danish laboratory [1]. Monkeypox is a zoonotic viral infection, first detected in humans in the Democratic Republic of Congo (DRC) in 1970 [2]. It is caused by an Orthopoxvirus belonging to the Poxvirdae family, known as monkeypox virus (MPXV). The symptoms caused by MPXV are very similar to the smallpox virus or variola virus such as fever, headache, muscle aches, backache, swollen lymph nodes, chills, and exhaustion [3]. Monkeypox virus is transmitted from one infected person to another by close contact interactions of body fluids, lesions, respiratory droplets, and household transmission [4]. The infection takes 6–13 days to develop symptoms in an infected person. There are many other similarities between the smallpox virus and MPXV. They are both double-stranded DNA viruses [1] and replicate entirely in the cytoplasm [5]. The genome length of these viruses is approximately 200 kb. However, while the WHO announced the worldwide eradication of smallpox in 1980 [6], MPXV cases in humans have continued to appear, and the virus has become endemic globally [7]. The monkeypox virus is related to variola and cowpox viruses in terms of the genome sequence and etiological perspective. The closeness is also reflected in the immune response, which is why the smallpox vaccine is known to have >85% protection against MPXV in humans [8].

There are two clades of MPXV based on its region of origin: clade I originated in the Congo Basin (Central Africa), and clade II originated in West Africa [9]. The recent human to human transmission of MPXV belongs to clade II. These lineages have further sublineages, such as A.1, A.2, B.1, B.2, and more [4]. The genomic sequences of individual West African-derived and Congo Basin monkeypox strains showed an overall nucleotide identity of only ∼95% across geographical groupings [10].

Through October 2022, there have been more than 72,000 cases of human monkeypox virus cased reported on the CDC website [11,12]. There are also a few cases of animals being infected by MPXV, where the animal was living in close proximity to humans [13]. Although the cases of monkeypox are rising throughout the world at an alarming rate, the severity of the infection is less. The reason for the sudden reappearance of this virus is uncertain.

We examined 1930 whole genome sequences of MPXV with a view to understanding the mutational patterns responsible for the current 2022 outbreak with respect to the initial MPXV sequences. We focused on the highly mutating genes in 11 Indian MPXV strains. In addition, we also investigated the effect of the above mutations on the MPXV protein structures.

## 2. Materials and Methods

### 2.1. Data Selection and Phylogeny

The number of cases was considered to be that reported by the CDC [11] and the WHO [12], and the graphs were produced in Graphpad Prism 9 and Microsoft Excel 2016. A FASTA database including 1930 MPXV whole genome sequences from GISAID Epipox [14] was built. The database included the sequences collected from the outbreak in 1962, the recent outbreak of 2022, and monkey host MPXV sequences from the Virus Pathogen Resource (VIPR) [15] (Supplementary File S1).

The genome-wide multiple sequence alignment of the 38-sequence database was performed using the Multiple Alignment using Fast Fourier Transform (MAFFT) algorithm version 7.505 [16]. A maximum likelihood tree with 100 bootstraps was constructed [17] using IQ-TREE [18] software version 2.0.

### 2.2. Genome Mutation Analysis

The number of insertions, deletions, and frameshift mutations in the Indian Strain (hMpxV/India/KL-ICMR-16-5316-553/2022|EPI_ISL_13953610|2022-07-13) were relative to the reference sequence (MPXV-M5312_HM12_Rivers) GenBank Accession MT903340 (NC 063383) used in pairwise alignment. The data were downloaded from Nextclade [19] after clade assignment, mutation calling, and sequence quality checks.

The substitution mutation rate for the outbreaks in 2021 and 2022 was calculated by the following equation [20]:

Mutation rate = (Mutation/lg/gs) × 100, (1)

where lg is the length of the dataset of the 1930 MPXV whole genome sequences downloaded from GISAID Epipox, and gs is the length of the reference genome sequence (MPXV-M5312_HM12_Rivers GenBank Accession MY903340), which was 197,209 bp in this case.

### 2.3. Computational Structural Analysis of the MPXV Proteins

The structures of the proteins involved in binding to the host receptors were generated using homology modeling via Swiss Modeling [21] to perform homology modeling of the proteins. The template structures of the proteins of vaccinia virus used were B14 (PDB ID: 2VVY, vaccinia virus E2 (PDB ID: 7PHY) and vaccinia virus protein 26 (PDB ID: 6A9S). The modeled structures were validated using the Ramachandran plot. The effects of the mutations on the protein structure were predicted by calculating the change in the Gibbs free energy using MAESTROweb [22]. The graphical representation of the monkeypox virus was created using BioRender.com, accessed on 20 October 2022.

## 3. Results

### 3.1. MPXV Genome Sequences from the Ongoing Outbreak Show a High Mutation Rate

**Figure 1:**
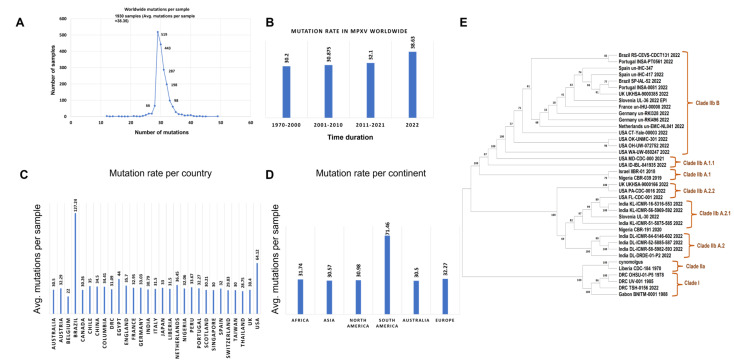
Distribution of the number of mutational events in the monkeypox genome sequences analyzed. (**A**) The average number of mutations per sample is 38.35 worldwide. (**B**) The mutations per sample of the MPXV outbreaks in 2021 and 2022. (**C**) The mutation events per country. (**D**) The distribution of the number of mutations for each sample per continent. (**E**) The maximum likelihood phylogenetic tree of the nucleotide sequences of the whole-genome MPXV strains selected from the collection date of 1962 to 2022 from the GISAID database across the world. The human MPXV genomes belong to clade IIb. The phylogenetic analysis was conducted using the maximum likelihood method with 1000 bootstraps.

We analyzed 1930 complete genome sequences from the GISAID database from previous outbreaks as well as the recent 2022 outbreak to calculate the mutations per sample in MPXV. Our analysis of the 1930 human MPV genomes highlighted a total of 74,556 amino acid level mutation events compared to the reference genome sequence MPXV-M5312_HM12_Rivers (GenBank Accession MT903340). The average number of mutations per virus sample worldwide was 38.35, as shown in Figure 1. The mutations per sample of MPXV was the highest at 38.63 in the 2022 outbreak, as compared to the outbreaks in 1970 at 30.2, 2007 at 30.87, and 2021 at 32.1. Amongst the nations with highest number of sequenced full viral genomes, the following countries had a significantly higher number of observed mutations per virus, when compared to the world’s average: Brazil (127.24) and the USA (64.12). On the other hand, the sequences from the following countries showed a significantly lower mutational burden: Belgium (22), Thailand (28.75), Switzerland (29.83), India (30.79), Singapore (30), Scotland (30.21), and Australia (30.5). One must bear in mind that some sampling biases may affect this comparison: for example, some countries generated the highest number of sequences in the early phases of the pandemic; thus, they may have observed fewer mutations. The mutation rate was calculated for 2021 and 2022 by using MPXV-M5312_HM12_Rivers as the reference genome, and we observed that the rate increased in the 2022 outbreak from 0.02 to 0.038 (Figure 1B). We analyzed the nature of each mutation, highlighting a strong prevalence of the Single-Nucleotide Polymorphisms (SNPs) over the short insertion/deletion events (indels) worldwide (Supplementary Figure S1).

To understand how divergent the distribution of MPXV throughout the world was, we retrieved 38 MPXV whole genome sequences, which included 29 sequences from the recent 2022 outbreak, four from the initial outbreak in 1970 in the Republic of Congo, and one animal-originated strain from *Macaca fascicularis* (Cynomolgus monkey) from North America. A phylogenetic tree based on the maximum likelihood clearly distinguished between clade I (sequences from the Democratic Republic of Congo) and clade II (sequences from the 2022 outbreak). Another observation in the study was the clustering of all the recent outbreaks in the European countries, the USA, and Africa into new lineages of clade IIb, the West African clade (Figure 1E). Our analysis showed that the Indian MPXV strains belonged to A.2 and A.2.1 based on the whole genome DNA sequence. The lineage B of clade II was more widely spread than lineage A. We observed that most of the sublineages of linage A and B had SNPs in the end regions, while the central region of the genome was quite conserved. We then focused on the variable regions present in the terminal regions of the MPXV genome sequences to understand the differences in the previous and recent outbreaks.

### 3.2. Variable End Regions of the MPXV Genome Show a Higher Number of Mutations

**Figure 2:**
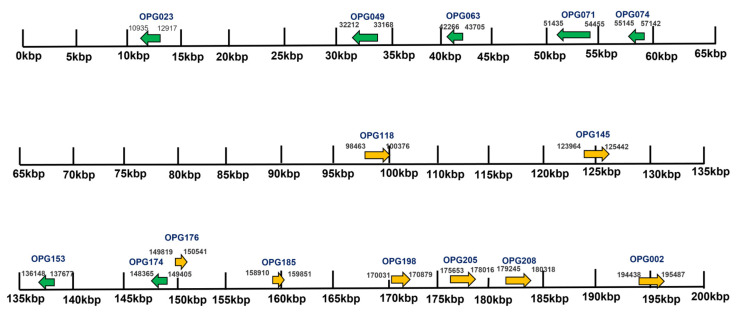
The mapping of the gene distribution in the Indian MPXV strain. The genes represented here in green (reverse) and yellow (forward) were highly mutated in the Indian HMPXV strain as compared to the reference sequence (MPXV-M5312_HM12_Rivers). These genes mostly occurred from 135 Kbp to 197 Kbp and from 10 Kbp to 60 Kbp in the terminal end region.

Of the 1930 total genome sequences, 11 Indian sequences were further selected consisting of five MPXV sequences from New Delhi, India, with no travel history and six sequences from Kerala, India, with recent international travel history. All the cases were symptomatic [23] and belonged to the A.2 lineage. We examined the proteins with the highest number of nonsynonymous mutations in all 11 sequences. The genes’ encoding for these proteins were mostly present near the terminal region from 10 Kbp to 60 Kbp towards the left terminal end and from 135 Kbp to 197 Kbp towards the right terminal end (Figure 2). The proteins, namely OPG023, OPG049, OPG063, OPG071, OPG074, OPG118, OPG005, OPG145, OPG153, OPG174, OPG176, OPG185, OPG198, OPG205, OPG208, and OPG002 were amongst those with the highest SNPs. The amino acid substitution by the SNPs were the most prevalent mutational events, followed by the silent SNPs and extragenic (mostly 5′UTR) SNPs. The silent events may not determine an immediate effect on the protein sequences, but they have repercussions, as they may change the codon usage and translation efficiency. Studies have demonstrated that silent synonymous polymorphisms can affect messenger RNA splicing, stability, and structure as well as protein folding [24]. In the case of the 5′UTR SNPs, the mutations may affect the transcription and replication rates of the virus [25].

The reference genome sequence available on NCBI was derived from the sample from ZAIRE-96-I-16 (GenBank Accession AF380138). The whole genome sequence was 198,858 base pairs long double-stranded DNA. Poxviruses have a common feature, as depicted in Supplementary Figure S2; the DNA strands are connected at their terminal to form a loop. The terminal end contains a set of inverted repeats present on both the left terminal end as well as the right terminal end. The central region of the genome coding for the DNA replication and assembly machinery is quite conserved as compared to the genes present in the variable regions of the terminal ends.

**Figure 3:**
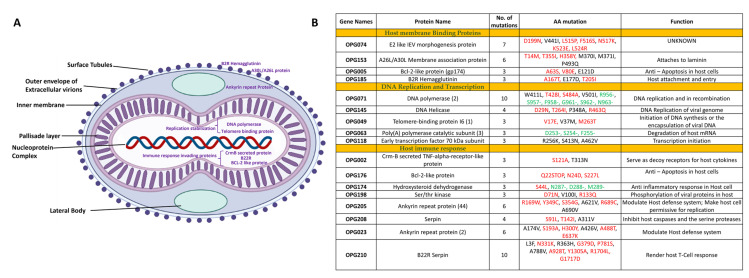
(**A**) Structures depicting the mutations in the Indian MPXV strains in comparison to the MPXV reference sequence. (**B**) List of the nonsynonymous mutations in the Indian MPXV sequences. The red denotes the mutations that are substitution mutations, and the green denotes the mutations that are deletion mutations.

The nonsynonymous substitutions, insertions, and deletion mutations in the genomes are listed in Figure 3B. We specifically estimated the mutation rate, the force that drives the evolution of viruses. Generally, the average mutation rates in RNA viruses are estimated to be about 100 times higher than those for DNA viruses. This rate is especially high because RNA viruses lack the sophisticated DNA repair mechanisms found in human and other animal cells [26]. The enzymes that occur in RNA viruses participate in copying viral genomes, which is a key reason for this difference. These enzymes lack the built-in capabilities to recognize the DNA damage that enzymes in most organisms have [26]. Though monkeypox belongs to the Orthopoxvirus family, which contains double-stranded DNA as genetic material, the MPXV managed to mutate relatively faster in the 2022 outbreak. We identified 17 proteins, which had more than three mutations and five proteins with more than six mutations in the Indian MPXV strain, which belonged to clade IIb and lineage A.2. The gene list included the BCL-2 like protein, the B22R family protein, the Ankyrin repeat Protein, the A30L protein, DNA polymerase, the CrmB secreted protein, serpin, DNA Helicase, and viral entry-related proteins. These genes were categorized into three groups, host cell binding, DNA replication and transcription, and host immune system invading groups based on their functions (Figure 3A).

The B22R family proteins were found to have 10 mutations making it the most highly mutated protein in the Indian monkeypox strain. The B22R proteins are known to render human T cells unresponsive to stimulation of the T cell receptor by MHC-dependent antigen presentation or by MHC-independent stimulation [27]. There were 11 Ankyrin repeat proteins in the genome of the monkeypox virus, starting from the N-terminal, across the genome until the C-terminal. The size of the Ankyrin motif proteins varied from 400 to 800 amino acid residues. The Ankyrin motif proteins are associated with protein–protein interactions [28]. Moreover, viral Ankyrin proteins are known to regulate B-cell activation [29]. Four out of the 11 Ankyrin proteins had at least six amino acid substitution mutations (Figure 3B). Based on this observation, it can be concluded that the variation in different copies of a gene, such as Ankyrin, is an important factor in regulating virus fitness. The substitution mutations in DNA polymerase (OPG071) and DNA Helicase enzymes (OPG145) might affect the DNA replication and recombination tendency of the viral DNA. The highest number of deletion mutations was observed in OPG034 (BCL-2 family protein), OPG071(DNA Polymerase), OPG210 (B22R), and OPG063 (Poly(A) polymerase catalytic subunit). The deletion in the terminal regions of the genome leads to progressive gene loss and remains a driving force behind the viral evolution [30]. Mutations in the envelope glycoproteins that help the virus bind to the proteins in host cells may lead to increased transmission and severity. The high number of mutations in the proteins involved in the host cell binding and immune system invasion suggests the ongoing modulation of the virus’ fitness.

### 3.3. Structural Analysis of the Mutations Suggests the Greater Fitness and Infectivity of the New Variants of MPXV

We further analyzed the effects of many of the mutations on the structure of the MPXV proteins. Computational structural analysis was carried out to understand the effects of the mutations on some of the highly mutated glycoproteins. Based on the host–pathogen analysis, a few key proteins such as the proteins A26L, BCL-2-like protein, and E2 were selected for structural analysis.

**Figure 4:**
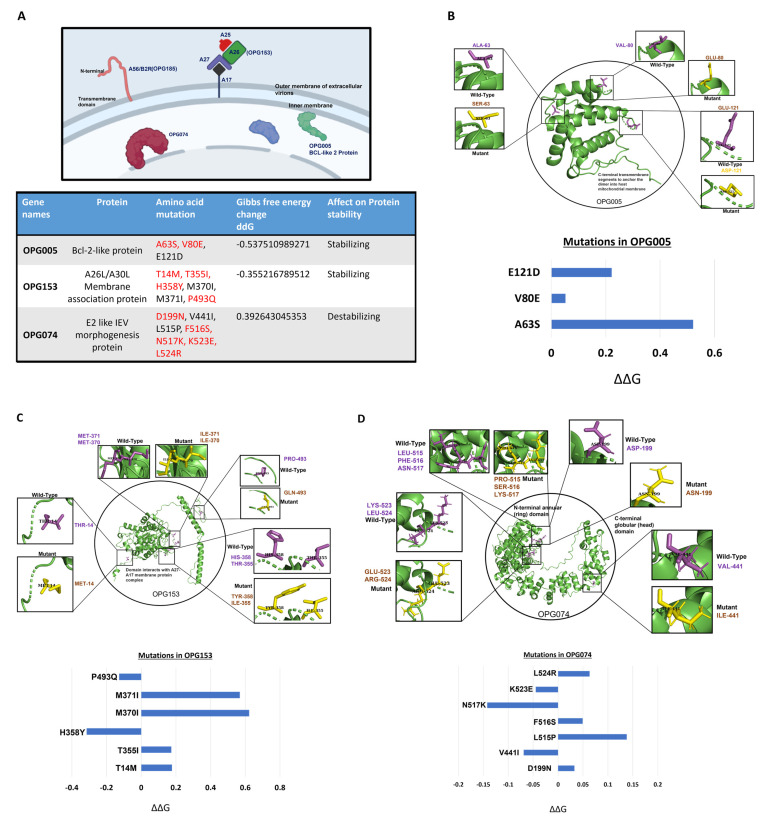
Structures depicting the mutations in the host cell binding viral proteins. (**A**) The localization of the OPG005, OPG153, and OPG074 viral proteins in the monkeypox virus structure. These three mentioned proteins are involved in host cell binding and were used to analyze the Gibbs free energy change due to the mutations in Indian strain: (**B**) OPG005; (**C**) OPG153; (**D**) OPG074. The Swiss Model structures show the position of the amino acid substitution mutation; pink shows the wildtype, and yellow shows the mutant-type amino acid.

Point mutations can have a strong impact on the protein stability. The amino acid substitutions due to these point mutations are represented in Figure 4 in protein structures generated using the Swiss Model. The modeled structures were validated using the Ramachandran plot. The secondary structure conformation of the modeled proteins can be viewed in Supplementary Figure S3. Out of 16 total, 11 mutations in OPG005, OPG153, and OPG074 were in the protein coding region, which generated 11 nonsynonymous amino acid substitutions. All 11 mutations could be high impact, as they substituted charged amino acid to uncharged, nonpolar to polar, nonpolar to charged, and vice versa impacting the structure and consequently the function of the protein. This point mutation induced a change in the thermodynamic stability of the protein structure, thereby affecting the function. As a final part of our analysis, we analyzed the effects of these mutations.

The structures of the proteins OPG005, OPG153, and OPG074, involved in binding to the host receptors were generated using the templates with PDB IDs 2VVY, 6A9S and 7PHY respectively. The structure of the OPG005 protein revealed that the monomeric polypeptide folded into eight helical segments and adopted a BCL-2 like domain (Figure 4B). A previous study showed that the substitution mutation in the BH3 domain of the protein led to tight binding to the host cell, as a result triggering key mitochondrial events associated with apoptosis [31]. OPG153 encodes for A26L/A30L, which enables the mature virions to enter through endocytic vesicles [32]. Moreover, A26 L binds to laminins and functions in cell attachment [33] (Figure 4A). A26 exists in a complex with three other viral proteins: A25, A27, and A17. The interaction of A26 with the A17 transmembrane protein is mediated through A27, and this provides the anchor for the localization of A26 on the surfaces of MVs [34]. The interaction of A26 and A27 is direct and stabilized by a disulfide bond. There were six nonsynonymous mutations in OPG153, which might affect its interaction with other proteins (Figure 4C). OPG074, while conserved across poxviruses, lacks an identifiable sequence homology to any other protein family. OPG074 is an E2-like Intracellular enveloped virion (IEV) morphogenesis protein and comprised a novel N-terminal annular (ring) and C-terminal globular (head) domains (Figure 4D). We identified seven amino acid substitutions in OPG074, which might affect the function of the protein.

In order to estimate the changes in the unfolding free energy due to point mutation, we used the sequences of the host cell binding proteins of the monkeypox virus. Stability analysis was conducted to predict whether the mutation was stabilizing or destabilizing by quantifying the ΔΔG (change in the Gibbs free energy). We analyzed in detail the three proteins involved in the viral entry and pathogenesis: OPG005, OPG153, and OPG074. The ΔΔG for the OPG153 and OPG005 were −0.53 Kcal and −0.35 Kcal, respectively, with good confidence of values 0.9 and 0.8, suggesting a stabilizing influence of these SNPs on the individual protein structures (Figure 4A). The ΔΔG for the multipoint mutations in OPG074 was 0.39 Kcal, which means it was destabilizing compared to the reference sequence. The protein–protein interaction and protein stability are greatly affected by both stabilizing and destabilizing mutations. Hence, it is important to study the mutational pattern of the monkeypox virus. Stabilizing mutations render a structural rigidity to the protein, which may provide increased protein–protein complex stability, which is host cell binding in the case of the monkeypox virus, resulting in greater fitness. However, as this Orthopoxvirus continues to evolve, new features may emerge or mutate alongside the genomic sequences, with clinical and pharmacological repercussions.

The ΔΔG analysis depicts the stability of the mutations of each amino acid. The mutations that yield an energy change (ΔΔG) higher than 0 destabilize the structure, and the mutations that bring an energy change (ΔΔG) lower than 0 stabilize it.

## 4. Discussion

The first monkeypox virus case was detected in the Democratic Republic of Congo (DRC) in 1970 in a 9-month-old baby. There were 406 cases between 1970 and 1990, 515 cases from 1990 to 1999, 65 cases from 2000 to 2009, and 428 cases from 2010 to 2020 in the MPXV-endemic African region [35]. In 2003, the first outbreak outside Africa happened in the USA after importing small mammals from Africa, namely prairie dogs (*Funiscuirus* spp, *Heliosciurus* spp, *Cricetomys* spp.). Twelve cases of person-to-person transmission of MPXV were detected in the USA; however, the severity of the infection was low. From January 2022 to October 2022, more than 72,000 cases of human-transmitted monkeypox virus have been confirmed [27], and more than 2510 complete sequences have been submitted to the viral database GISAID [14]. The virus outbreak has become a pandemic in many parts of the USA and Europe, where it was earlier not endemic. To understand the fast-progressing transmission of the MPXV infection, it is important to monitor the genetic features of the evolving virus by studying the mutation rate.

In this study, we analyzed the mutation rate of the monkeypox virus in the previous and recent outbreaks across the globe, and we concluded that the mutation rate was relatively higher in the 2022 outbreak. Unlike COVID-19, it is not highly contagious and is a poor spreader. It has been transmitted in age groups of mostly 15–50 years old, but recently in July 2022, the WHO reported more than 80 children across several countries were infected. Our data demonstrated that the evolution of the MPXV virus is ongoing in human-to-human transmission. The monkeypox virus has mutated over time, resulting in genetic variations in the circulating strains, also called lineages. We report that the Indian MPXV strain belongs to the A.2 lineage and clusters away from most other strains spread across the world. Our results highlighted the effects of the mutations in the MPXV protein structures and functions for the proteins involved in host cell attachment. These genetic variations may impact the virus’s properties such as transmission or the severity of symptoms in infected individuals.

Since monkeypox is a viral zoonosis, the human and animal health sectors need to coordinate their interventions. There is a possibility that the monkeypox virus has established itself into a suitable reservoir host, which is currently unknown. If such a reservoir host exists, the virus can transmit to other animal species as well [36]. Constant monitoring of mutations will also be pivotal in tracking the movement of the virus between individuals and across geographical areas. Tracking viral evolution will be possible by constantly monitoring the MPXV genomic sequences.

## Data Availability

All data and analysis methodologies are contained in the manuscript. Any additional data requests can be addressed to the corresponding authors.

## Supplementary Materials

The following supporting information can be downloaded at: https://www.mdpi.com/article/10.3390/v15010127/s1, Figure S1: Spreading of MPXV worldwide; Figure S2: The mapping of gene distribution in MPXV strains sequences; Figure S3: Ramchandran plots for OPG005, OPG153 AND OPG074; File S1: Monkey host MPXV sequences from the Virus Pathogen Resource (VIPR).
