# Supplementary material for: Phylogenomic and Structural Analysis of the Monkeypox Virus Shows Evolution towards Increased Stability
Source: Viruses. 2022 Dec 31;15(1):127. doi: 10.3390/v15010127 (PMC9864997; doi:10.3390/v15010127)

Supplementary Figures:

Supp Figure S1. **Spreading of MPXV wordwide. A) MPXV outbreaks before 2022. B) MPXV outbreaks in 2022.**

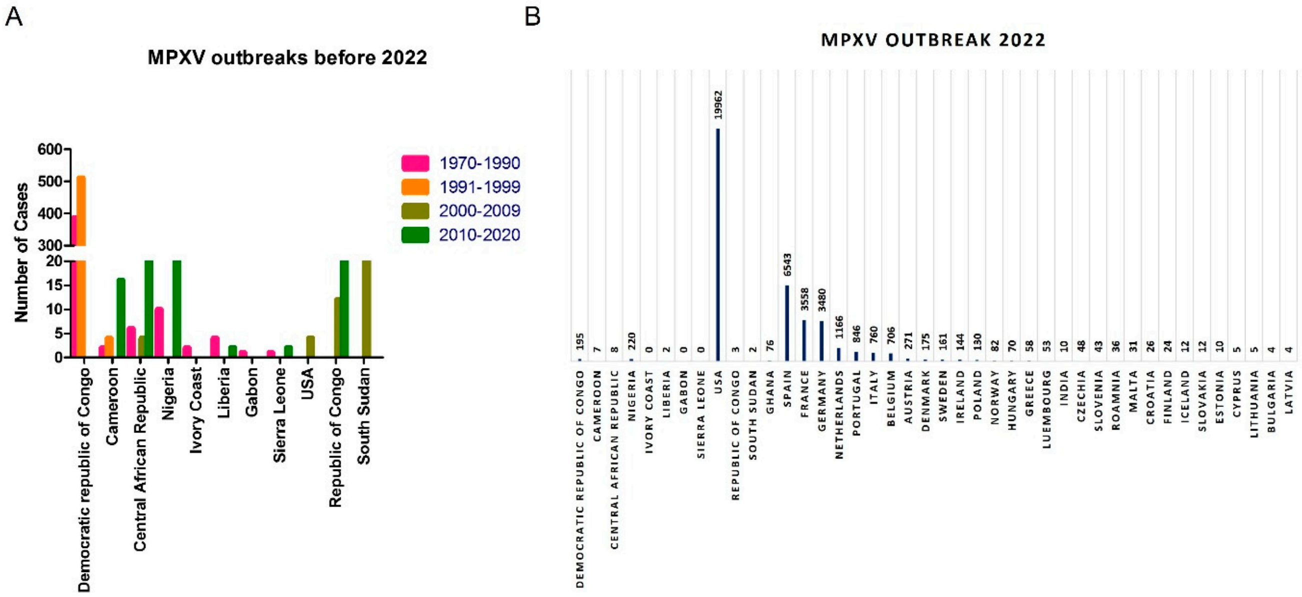

Supp Figure S2. The mapping of gene distribution in MPXV strains sequences

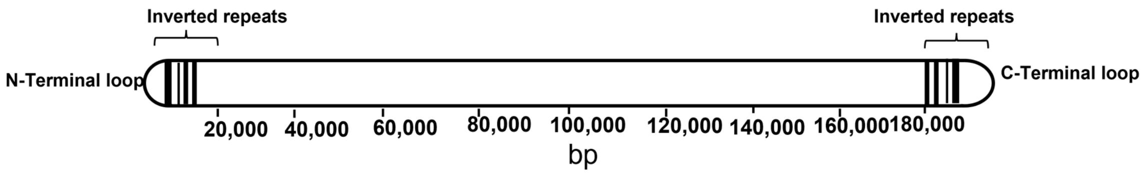

Supp Figure S3. Ramchandran plots for OPG005, OPG153 AND OPG074

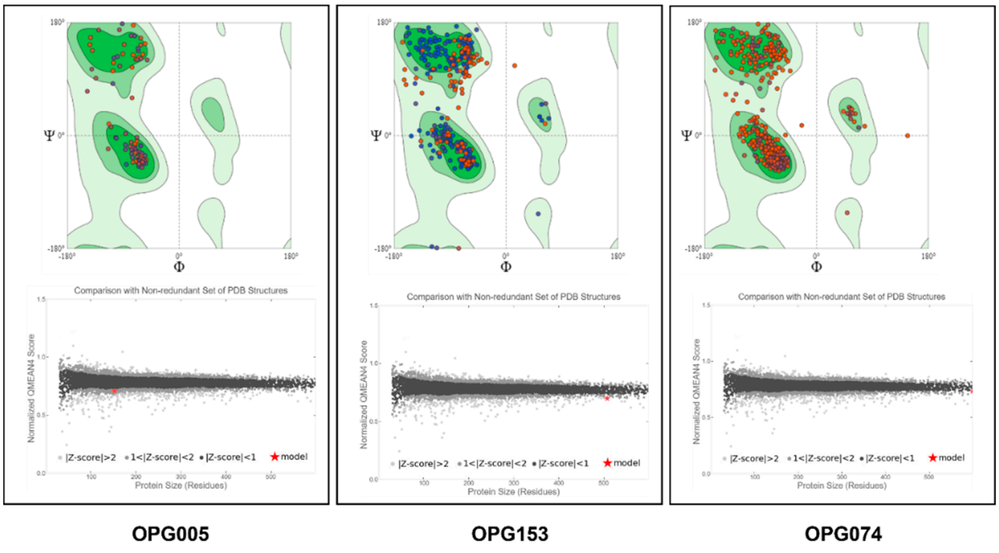

Supplement: Supplementary file 1 [file viruses-15-00127-s001.zip › Supplementary Figures.pdf]
